# Changes in *Lolium perenne* L. rhizosphere microbiome during phytoremediation of Cd- and Hg-contaminated soils

**DOI:** 10.1007/s11356-023-25501-y

**Published:** 2023-02-13

**Authors:** Juan F. Saldarriaga, Julián E. López, Laura Díaz-García, Carolina Montoya-Ruiz

**Affiliations:** 1 Dept. of Civil and Environmental Engineering, Universidad de los Andes, Carrera 1Este, #19A-40, 111711 Bogotá, Colombia; 2 Facultad de Arquitectura E Ingeniería, Institución Universitaria Colegio Mayor de Antioquia, Carrera 78 # 65 – 46, 050034 Medellín, Colombia; 3 Department of Chemical & Biological Engineering and Advanced Biomanufacturing Centre, University of Sheffield, Sir Robert Hadfield Building, Mappin Street, Sheffield, S1 3JD UK; 4 Facultad de Ciencias, Universidad Nacional de Colombia, Sede Medellín Calle, 59A #63-20, 050034 Medellín, Colombia

**Keywords:** Microbiome, *Lolium perenne* L., Hg, Cd, Phytoremediation

## Abstract

The contamination of soil and water by metals such as mercury (Hg) and cadmium (Cd) has been increasing in recent years, because of anthropogenic activities such as mining and agriculture, respectively. In this work, the changes in the rhizosphere microbiome of *Lolium perenne* L. during the phytoremediation of soils contaminated with Hg and Cd were evaluated. For this, two soil types were sampled, one inoculated with mycorrhizae and one without. The soils were contaminated with Hg and Cd, and *L. perenne* seeds were sown and harvested after 30 days. To assess changes in the microbiome, DNA isolation tests were performed, for which samples were subjected to two-step PCR amplification with specific 16S rDNA V3-V4 primers (337F and 805R). With mycorrhizae, changes had been found in the absorption processes of metals and a new distribution. While with respect to microorganisms, families such as the *Enterobacteriaceae* have been shown to have biosorption and efflux effects on metals such as Hg and Cd. Mycorrhizae then improve the efficiency of removal and allow the plant to better distribute the absorbed concentrations. Overall, *L. perenne* is a species with a high potential for phytoremediation of Cd- and Hg-contaminated soils in the tropics. Inoculation with mycorrhizae modifies the phytoremediation mechanisms of the plant and the composition of microorganisms in the rhizosphere. Mycorrhizal inoculation and changes in the microbiome were associated with increased plant tolerance to Cd and Hg. Microorganism-assisted phytoremediation is an appropriate alternative for *L. perenne*.

## Introduction

Heavy metal contamination has caused serious environmental problems, generating degradation in ecosystems, as well as direct damage to human health. Of the 118 elements known to man, 98 are metals, which have played a fundamental role in the development of civilizations. The problem has focused on the rapid demographic growth and industrialization that have led to serious problems of pollution and deterioration of the environment, especially in developing countries (Wu et al. 2022). Among the metals of greatest environmental concern are lead (Pb) and mercury (Hg), followed by beryllium (Be), barium (Ba), cadmium (Cd), copper (Cu), manganese (Mn), nickel (Ni), tin, (Sn), vanadium (V), and zinc (Zn) (Gan et al. 2017; C. Li et al. 2022a, b, c; Singh et al. 2020). Industrial and mining activity releases metals such as mercury and cadmium into the environment, which are very harmful to human health and most living forms. Anthropogenic activities pollute the soil, and these metals bioaccumulate in plants, increasing their danger; later their concentration in living beings biomagnifies, so the ingestion of contaminated plants or animals can cause intoxication symptoms (Budianta 2021; Yang et al. 2022). Despite abundant evidence of these harmful effects on health, exposure to heavy metals continues and can increase due to the lack of a consensual and concrete policy in different countries, especially in developing countries. Mercury is still used extensively in the gold mines of Latin America, while cadmium is found in many fertilizers and pesticides that are applied daily in agricultural areas. Cadmium (Cd) and mercury (Hg) accumulation in soils has been rapidly increased due to natural (e.g., sediments) and anthropogenic (e.g., mining and agriculture) process (Y. Li et al. 2022a, b, c; Liu et al. 2020; Yuan et al. 2022). These heavy metals are non-biodegradable.

Countries located in the tropical zone contribute approximately 30% of Hg pollution worldwide. Especially, South America contributes 2% to this phenomenon (Singh and Kumar 2020). Colombia is the third country in the world with the highest mercury pollution, after China and Indonesia, emitting approximately 75 tons per year into the environment (MADS 2014). In Colombia, there are areas with a high concentration of mercury reported up to 340 μg/m^3^ in the air (300 times higher than the World Health Organization guideline for maximum public exposure to mercury vapor) (MADS 2014). Even in some urban areas of Colombian mining municipalities such as Segovia, mercury concentrations vary between 40,000 and 80,000 ng/m^3^, far exceeding the permissible value of 10 ng/m^3^ (UNIDO 2017). The average level of cadmium in soils has been located between 0.07 and 1.1 mg/kg, with a natural base level that would not exceed 0.5 mg/kg. Some soils can have higher levels of cadmium because the rocks that formed them had cadmium in their composition. For example, phosphate rocks, which are the raw material for all phosphate fertilizers, contain levels of heavy metals that vary according to their geographical origin but are generally higher than the first in the earth’s crust (Wiggenhauser et al. 2019). Cadmium remains in a significant proportion in industrial fertilizers and is subsequently applied to the soil together with phosphorus (Jensen and Mosbæk, 1990).

The increase in these pollutants in the soil has played an important role in recent years since agricultural ecosystems and mining areas have been affected mainly by the increase in population (Eliana Andrea et al. 2019). Consequently, the health of the soil determines the stability and balance of ecological systems. The increase of metals such as Hg and Cd in soils can exceed their buffering capacity, leading to possible spread throughout the environment and being able to enter the food chain (Biswas et al. 2020; W. Li et al. 2022a, b, c). Therefore, a soil remediation approach that involves living organisms (plants and microorganisms) with high adsorption capacity and high availability is necessary. Among these processes is phytoextraction, which is considered a commercial phytoremediation method with greater projection in the coming years (Zhao et al. 2019). Studies on the remediation of metals such as Hg and Cd have focused on removal processes of these from plants (Cruz et al. 2021, 2019; Leudo et al. 2020). *L. perenne* (ryegrass) is a grass native to Europe, Asia, and North Africa and is now widely distributed throughout the world, including the Americas and Australia. This plant has been extensively studied due to its great response to abiotic stress exposure of metals such as Hg and Cd (Cruz et al. 2021). Some authors have verified its phytoremediation capacity of heavy metals individually of Cd and Hg (Cruz et al. 2021, 2019). On the other hand, studies have also been carried out in which this plant is placed in symbiosis with mycorrhizae, finding promising results in the elimination of Hg in soils (Leudo et al. 2020). Regarding the molecular response, alterations of the *GST* gene have been observed, which is important because this gene encodes proteins that can help eliminate toxins from the plant (Cruz et al. 2021).

However, it has been argued that of all the technologies used for metal removal, bioremediation using microorganisms has gained the most attention, due to its better ability to resist rapid mutation and environmental evolution, compared to plants and animals (Zhao et al. 2019). By understanding the process and content of genetic information within a contaminated sample, 16S amplicon sequencing allows us to understand the broad changes in community diversity over time, which combined with metagenomics increases the resolution and sensitivity of understanding microbial communities (Poretsky et al. 2014). For this reason, different amplicon sequencing and metagenomic analyses have been developed with the evaluation of the collective genomes and the biosynthetic machinery of the soil microflora (Handelsman et al. 1998). Gene-directed metagenomics has also been developed to investigate metal-contaminated soils using polymerase chain reaction–based targeting in conjunction with pyrosequencing. Also, deep sequencing of rRNA genes and functional regions has been shown to help in the development of new bioremediation strategies (Bell et al. 2011; Brennerova et al. 2009; Huang et al. 2022; Iwai et al. 2010; Malla et al. 2018). It has been proven that from metagenomic analysis, it is possible to identify indicator species that are specific for certain contaminants that may be targeted, and with this, effective ways to modify or resist adverse environmental conditions could be identified (Huang et al. 2022). For this reason, the main objective of this work had been the metagenomic evaluation of a phytoremediation process of soils contaminated with Hg and Cd by means of *L. perenne*-mycorrhizae and the changes in the soil microbiome.

## Materials and methods

### Plant and arbuscular mycorrhizal fungi inoculum

Certified *L. perennial* seeds referenced as Rye Grass Bestfort plus (CEBA, Bogotá, Colombia) were used. Before being used, they were washed with deionized water and 10% KOH solution. The reference Tierra Bona (Fercon S.A., Cali, Colombia) was used for the soil, and one commercial preparation was used to form the mycorrhizae. The “MICORRIZAR Biological Soil Inoculant” (AGROTECNIA LTDA, Bogotá, Colombia), which is roots colonized with a mixture of spores of the genera *Glomus* sp., *Acaulospora* sp., *Entrophospora* sp., and *Giaspora* sp. The association test was evaluated in previous work (Leudo et al. 2020). The researcher found that this brand was the only ones that achieved an association with the root of the plants and the least amount of inoculant.

### Soil sampling and characterization

Soil samples were collected from top layer (0–20 cm) following a non-systematic sampling scheme in zigzag. The samples were bulked together to obtain an average soil sample that was air-dried, sieved (< 2 mm), and homogenized. Then, composite sample was used for determination of soil properties. Soil pH and electrical conductivity (EC) were measured in deionized water (DW) used 1:2 soil/water ratio. Particle size distribution was determined by hydrometer method. Organic carbon was measured by Walkley–Black method. Total N was determined for Kjeldahl method. Al, Ca, Zn, Fe, Mg, Mn, K, Na, Cd, and Hg were extracted with aqua regia method, and pseudo total concentration was measured using inductively coupled plasma optical emission spectrometry (ICP-OES) in an ICP-OES Thermo Scientific™ ICAP6500 DUO kit (Thermo Scientific, Waltham, MA, USA) equipment. Soil properties are summarized in Table 1.

**Table 1:** Soil properties

Parameter	Value
Al (mg/kg)	43.35 ± 1.23
Ca (mg/kg)	7490.00 ± 10.26
Zn (mg/kg)	61.50 ± 3.12
Fe (mg/kg)	19.57 ± 1.25
Mg (mg/kg)	1795.00 ± 12.69
Mn (mg/kg)	778.00 ± 13.64
K (mg/kg)	1457.00 ± 18.24
Na (mg/kg)	322.00 ± 9.87
Cd (mg/kg)	0.57 ± 0.03
Hg (mg/kg)	0.61 ± 0.01
N (%)	0.054 ± 0.00
Total organic carbon (TOC) (%)	4.50 ± 0.21
pH	6.07 ± 0.32
EC (μS/cm)	4.30 ± 0.27
Sand (%)	15
Silt (%)	30
Clay (%)	55

### Soil contamination

The Cd and Hg concentration in spike solution was selected based on total soil Cd and Hg (Arias Espana et al. 2018). The spiking solution was spread onto the soils while mixing thoroughly using a plastic spatula. After spiking, average pseudo total Cd and Hg concentration in soil samples were 3.06 (± 0.12) and 3.12 (± 0.09) mg kg^−1^, respectively, reaching the targeted concentrations. All treatments were brought to 80% WHC with DW and allowed to further equilibrate for 8 days.

### Rhizobox experiment system

Four treatments were evaluated in duplicate according to previous studies (Cruz et al. 2021, 2019; Leudo et al. 2020). Considering that the best ratio was one part of mycorrhiza to three parts of soil, the quantities of each are shown in Table 2.

**Table 2:** Treatments used for the metals used phytoremediation process

Treatment	Amount of soil (g)	Amount of mycorrhiza (g)
B (control)	1000 *	-
BM (control with mycorrhizae)	667	333
H (mercury)	1000	-
HM (mercury with mycorrhizae)	667	333
Cd (cadmium)	1000	-
CdM (cadmium with mycorrhizae)	667	333

* Rhizobox capacity

In each rhizobox (with dimensions of 20-cm long, 10-cm wide, and 13-cm high), 200 seeds of *L. perenne* were sown and harvested at 30 days. The treatments used in the rhizobox culture were control (B), control with mycorrhizae (BM), mercury (H), cadmium (Cd), mercury with mycorrhizae (HM), and cadmium with mycorrhizae (CdM). During the 30 days of growth, the seedlings were irrigated with distilled water (207.5 mL) to maintain humidity at 70%. The watering was done on Mondays and Fridays at 8 am; likewise, the laboratory conditions were a relative humidity of 52%, with an average temperature of 20/16 °C, and the plants were exposed to a 12 h/12 h photoperiod. After 30 days, when the seedlings were collected, 95% of the plants showed uniformity in growth, and there were two biological samples (*n* = 2) per modality to carry out the analyses: molecular and heavy metal.

### DNA isolation

Per each culture, 500-mg soil samples were collected in 1.5 mL microcentrifuge sterile tubes and stored at − 80 °C until the DNA isolation using the Mag-Bind® Environmental DNA isolation kit (Omega Bio-tek, GA, USA) was done. Then, DNA was quantified using nanodrop to 260-nm absorbance and the 280/260 ratio was determined. Frozen pure DNA samples were sent to AMB company (Richmond, Canada) where the quality of the samples was determined by Qubit DNA Assay Kit. The samples were subjected to two-step PCR amplification with 16S rDNA V3-V4 specific primers (337F and 805R) followed by adapter addition, with a sequence coverage of 50 × . All the samples were successfully amplified, and the quality of the library preparation was measured using the Agilent 2100 Bioanalyzer. Finally, the paired-end sequencing was carried out in Illumina MiSeq with 2 × 300 bp strategy.

### Quality assurance and quality control

To assure the reliability of the results, quality assurance and quality control protocols were included. For all soil and plant samples, duplicates were included to evaluate reproducibility. The coefficients of variation for each set of duplicate reference samples ranges from 0.9 to 10.1% (average 5.2%) and from 0.3 to 12.0% (average 5.5%) for Hg and Cd in plant and soil samples. Certified reference materials (CRMs) were included in each digestion/extraction batch for quality assurance. The CRMs and certified Cd and Hg were ERM-CC141 loam soil, NIST 2709 A San Joaquin soil, NIST SMR 1573a Tomato leaves, and NIST SRM 1570a Spinach leaves. The recoveries of Cd and Hg calculated relative to the certified concentration were 88.45–105.61% for soil and 93.44–98.70 for leaves. All reagents were of analytical grade, and the glassware was soaked in an acid bath (3% v/v HNO3) overnight prior to use.

### Data analysis

For the data obtained from the shoot and root measurements, the Shapiro–Wilk normality test was applied. From these results, it has been found that the data did not present a normal distribution; comparisons of the lengths between treatments were made using the Kruskal–Wallis test. For both cases, a significance level of 0.05 was determined, and both tests were carried out with the help of the software Minitab 2019®. Sequencing data was processed using Quantitative Insights into Microbial Ecology (QIIME2) software (Bolyen et al. 2019). Amplicon sequence variants (ASVs) were determined using the deblur pipeline (Amir et al. 2017). Taxonomy assignment was done by aligning the reads against SILVA SSU database 138 integrated in QIIME v2020.11 (Parks et al. 2018). To calculate differential relative abundances, the DESeq2 (Bioconductor) add-on to the Phyloseq package was used using the R studio software. Statistical differences in alpha diversity were calculated using the pairwise Kruskal–Wallis test in QIIME2. PERMANOVA was used to perform multivariate analysis of beta diversity metrics.

## Results and discussion

### Phytoremediation of Cd- and Hg-contaminated soil

**Fig. 1:**
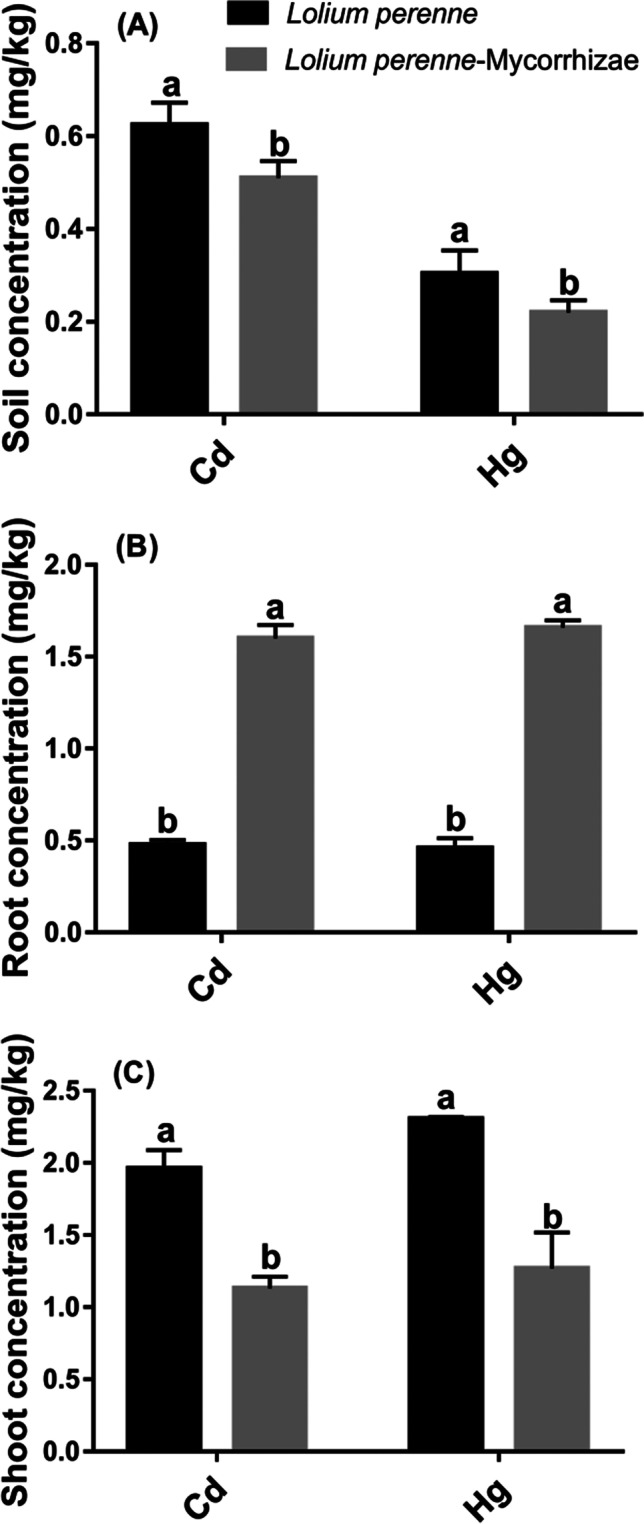
Cadmium (Cd) and mercury (Hg) concentration in (a) roots and (b) shoot of *L. perenne*. (**A**) Soil concentration, (**B**) Root concentration, and (**C**) Shoot concentration. Data (means ± SE, *n* = 3) followed by different subscripts denotes significant differences between treatments at *p* < 0.05 according to Tukey’s HSD. Treatments: *L. perenne* and *L. perenne*-mycorrhizae

A total of 460 ASVs were obtained, and data with relative abundance > 2% were retained for further ecological parameter analysis. The results show a higher decrease in Hg concentration compared to Cd concentration in soils, whether mycorrhizal or not (Fig. 1a). Cd concentration decreased on average 5.3-fold, while Hg concentration decreased on average 11.5-fold compared to the initial concentration in soil (Table 1). The presence of mycorrhizae generated significant changes in the bioaccumulation of Cd and Hg (Fig. 1b and 1c). On average, root Cd and Hg concentrations were fourfold higher in the mycorrhizal treatment compared to the non-mycorrhizal treatment. Cd and Hg concentration in shoots was on average twofold lower in the treatment with mycorrhizae than in the treatment without mycorrhizae. A higher removal of Cd and Hg in the soil for treatment with mycorrhiza showed that the presence of this fungus mitigates the toxicity of the pollutants and increases the tolerance of the plant to this metals (Lounès-Hadj Sahraoui et al. 2022). Therefore, its bioaccumulation capability is improved. Thus, mycorrhiza-assisted phytoremediation is shown to be a promising alternative for *L. perenne* as eco-sustainable technique to control and mange soil pollution.

**Fig. 2:**
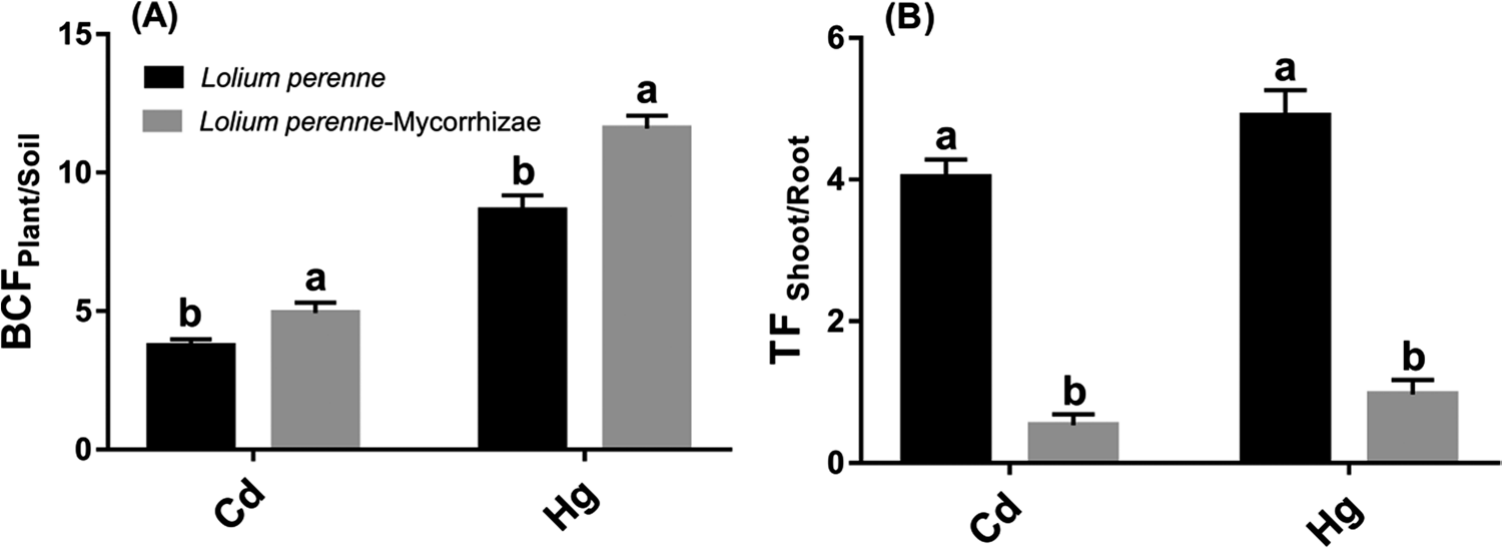
Cadmium (Cd) and mercury (Hg) bioconcentration factor (*BCF*_Plant/Soil_) (**A**) and translocation factor (*TF*_Shoot/Root_) (**B**) of *L. perenne*. Data (means ± SE, *n* = 3) followed by different subscripts denotes significant differences between treatments at *p* < 0.05 according to Tukey’s HSD. Treatments: *L. perenne* and *L. perenne*-mycorrhizae

The presence of mycorrhiza had a significant effect on *BCF*_Plant/Soil_ and *TF*_Shoot/Root_ factors (Fig. 2a and 2b). In the *L. perenne-mycorrhizae* treatment, the *BCF*_Plant/Soil_ increased 1.1-fold for Cd and 1.6-fold for Hg, compared to the non-mycorrhizal plant. Mycorrhization generated an 8- and fivefold decrease in TF for Cd and Hg, respectively. In line with the results of this study, mycorrhizae had been reported to influence the fate of metals in the rhizosphere through various phytotechnologies (Lounès-Hadj Sahraoui et al. 2022), in this case, phytostabilization and phytoextraction. According to the results, the presence of mycorrhizae modified the phytoremediation mechanism of *L. perenne* from a phytoextraction process to a phytostabilization process. The results without mycorrhiza are like those found in other studies with *L. perenne* (Huang et al. 2018). In the removal of Pb, it has been observed that the bioaccumulation of the metal occurs in greater quantity in the shoot, while in the root this concentration is lower (Huang et al. 2018).

The phytoremediation capability of *L. perenne* is limited for metals such as single Hg and Cd or a mixture of metals (Gavrilescu 2022; Li et al. 2020; Zhang et al. 2019). Therefore, as mentioned above and as shown in Fig. 1, the application of mycorrhizae stimulates the bioaccumulation of metals in the plants, changing the distribution of metals, helping their uptake and the increase in metal removal.

### Difference in the microbial community

Samples were successfully sequenced. Reads per sample, phred quality score (Qscore), and GC obtained are listed in Table 3. Qscore 20% was higher than 91 and GC% was close to 50 across the samples, indicating the good quality of the sequencing process. In addition, rarefaction curves achieved the plateau for all samples suggesting that the sequencing depth was high enough to analyze the diversity.

**Table 3:** Number of reads per sample and the phred quality score (Qscore) and GC contained

Sample	Treatment	Number of read	R1 Q20%	R2 Q20%	R1 Q30%	R2 Q30%	R1 GC%	R2 GC%
S1	Hg + M	55,965	99	98	74	89	53	53
S2	Cd + M	197,104	98	95	69	59	51	51
S3	B	95,013	99	97	89	77	57	56
S4	Hg	184,749	98	91	50	37	49	50
S5	M	183,458	99	95	75	63	51	51
S6	M	191,550	99	96	75	64	51	51
S7	Cd	121,782	99	97	90	72	57	56
S8	Hg	167,614	99	97	87	78	57	56
S9	Hg + M	66,639	99	98	80	85	56	56
S10	B	222,212	99	97	90	74	58	57
S11	Cd + M	144,498	99	97	86	73	56	55
S12	Cd2	107,460	99	96	90	64	54	54

**Fig. 3:**
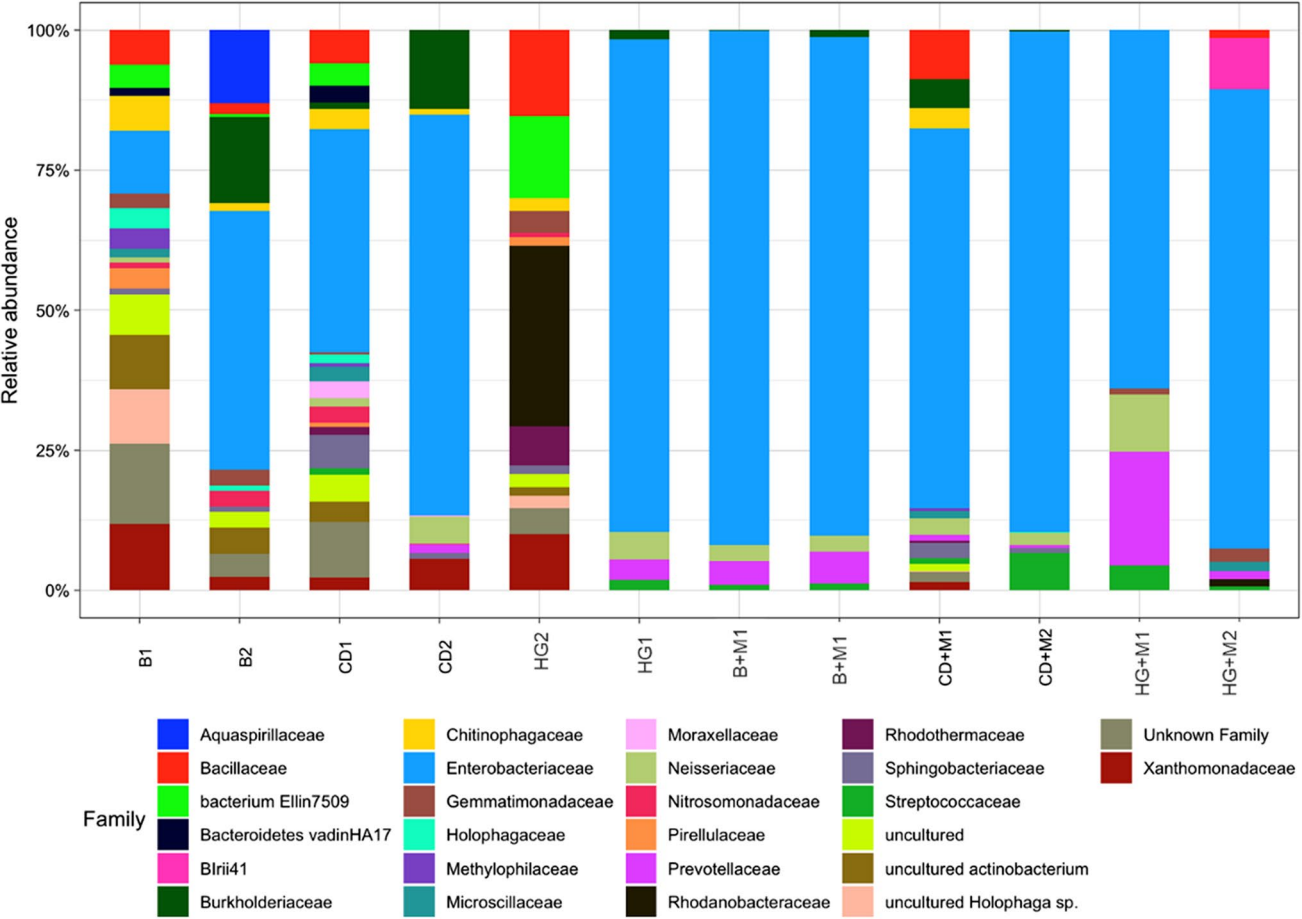
Relative abundance of bacteria families for the different treatments and replicas. The 16 s amplicon sequences variant were plotted with a cutoff of 2%. In the X axis numbers indicated the replica (1, first and 2, second) and the treatments (B, control without contamination; CD, cadmium contamination; H, mercury; B + M, control-mycorrhiza without contamination; CD + M, cadmium contamination with mycorrhiza; HG + M, mercury contamination with mycorrhiza)

Based on the taxonomic affiliation of ASVs, bacterial relative abundance between the treatments is illustrated in the stacked bar chart (Fig. 3). It is noteworthy, that for most sequences were possible to assign in family level, being the total abundance of bacterial families lower in the soil treated with mycorrhiza. This is probably due to an effect of mycorrhizal addition on the composition of the microbial community, in a similar way as has been suggested by other authors (Fan et al. 2020; Zhou et al. 2022). However, the variability between biological replicas and the limited number of samples make it difficult to observe statistical differences among the groups and find any conclusion about this phenomenon. For all treatments, except one replicate with mercury without mycorrhiza (Hg), the most abundant bacterial family was *Enterobacteriaceae*, indicating the organic composition of the soil (Cernava et al. 2019).

**Fig. 4:**
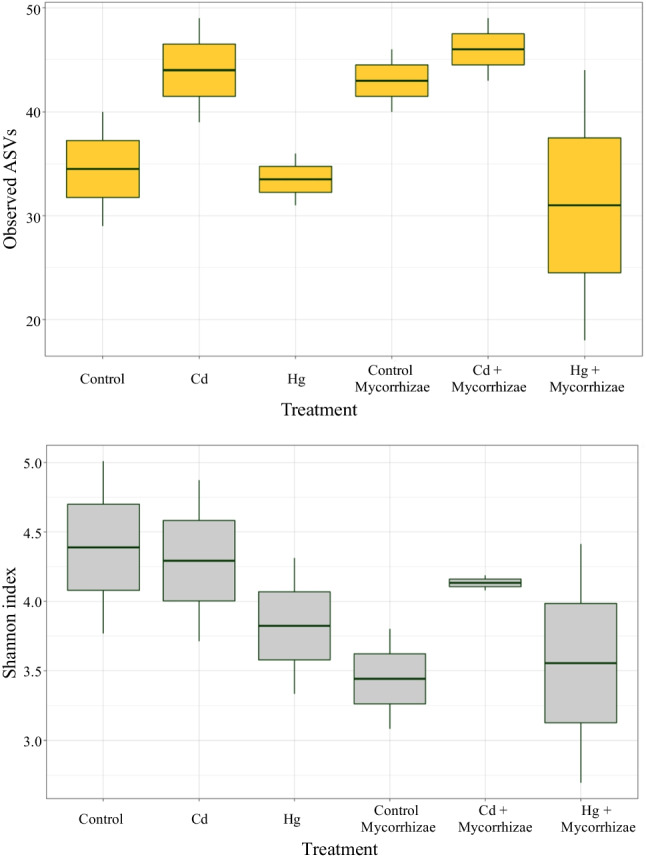
Comparison of alpha diversity among the treatments: **a** the ASV observed among the treatments, **b** the Shannon index among the treatments

The estimation of the alpha diversity was performed and is presented in Fig. 4. High values of Shannon index on the control sample, indicated higher riches in the microbial community in comparison with the contaminated soil. This observation could indicate an effect of these contaminants on soil microbial communities, where some populations may be susceptible to the presence of these contaminants, despite the known bioaccumulative effect of *L. perenne* and its ability to remove contaminants.

Statistical comparison between the number of observed ASVs and the Shannon index was performed using the DESseq2 statistical package and Kruskal–Wallis test. No statistical differences (*p*-value > 0.05) were found between treatments in observed ASVs and Shannon index (Fig. 4). These results indicated that there are not significant differences in the diversity of the whole bacterial community. However, the DESeq2 analysis found that there are some taxa in the mycorrhizal-only soil with a significantly different (*p*-value < 0.05) frequency than the control, suggesting the potential for mycorrhizal treatment to modify soil microbial composition (Fan et al. 2020).

In other studies with *L. perenne*, it has been found that its roots form arbuscular-type mycorrhizae (Leudo et al. 2020), which, according to some authors, makes them unique model systems for the study of interactions between plants and microorganisms (Gómez-Sagasti et al. 2021). In this study, it has been proven that mycorrhizae favor the presence of other microorganisms that help in the performance of metal uptake by the roots. In contrast, the rhizobacteria found in this study, which are resistant to metals, have in some cases improved plant growth (Fig. 1 and Fig. 2) despite the presence of metals. (Breton-Deval et al. 2022). According to other authors, it can be asserted that the availability of nutrients is being increased, through the biotransformation or sequestration of metals, across the modification of the metal-plant interaction (Breton-Deval et al. 2022; Gupta et al. 2017; Novo et al. 2018). Therefore, the roots that are interacting within the niche with innumerable microbial communities influence the growth of the plant. The presence of mycorrhizae allows the increase of communities of microorganisms and significantly improves resistance to stress. An improvement has been observed in the absorption of metal concentrations (Fig. 2) and their distribution between the root and the shoot (Kumawat et al. 2022; Panke-Buisse et al. 2015). Similarly, it allows the joint formation of the plant root microbiome (Kumawat et al. 2022; Philippot et al. 2013; Rich et al. 2017). The results presented in this study give an overview of the enormous diversity of species, the amazing interactions, and the complex structure within the rhizosphere. This permits an approximation to the understanding of the biological character of the root system and its microbiota in the process of phytoremediation of Hg and Cd from *L. perenne* (Hacquard 2016; Kumawat et al. 2022).

The design of this study made it difficult to observe other differences with the DESeq 2 analysis, probably due to the few replicates taken per treatment, because of the high costs of the sequencing process. Nonetheless, taxa with a frequency 100 sequences higher than the control soil were calculated (taxon count). *Shigella sonnei*/*Escherichia fergusonii* were found overexpressed in all contaminated soil with and without mycorrhizae, *Streptococcus mitis* in the Cd and mycorrhizae treatment (Cd + M), *Delftia tsuruhatensis*/*Delftia lacustris* in the (Cd) treatment, and *Neisseria cinerea*/*Neisseria perflava* in (Hg). In addition, *Prevotella melaninogenica* and *Vulgatibacter incomptus* were enriched in soil with mycorrhizae (M) (Table 4).

**Table 4:** The taxon with frequency 100-fold higher in comparison with the control

Taxonomy SILVA	Taxonomy blast	Percentage identity	*p*-value	Taxa count	Condition
*Streptococcus*	*S. mitis*	99,78	> 0,05	208	Cd + M
*Escherichia-Shigella*	*S. sonnei/E. fergusonii*	99,78	> 0,05	490	Cd + M
*Escherichia-Shigella*	*S. sonnei/E. fergusonii*	99,78	> 0,05	2653	Cd + M
*Escherichia-Shigella*	*S. sonnei/E. fergusonii*	99,78	> 0,05	138	Cd + M
*Escherichia-Shigella*	*S. sonnei/E. fergusonii*	99,78	> 0,05	182	Cd + M
*Escherichia-Shigella*	*S. sonnei/E. fergusonii*	100	> 0,05	153	Cd + M
*Escherichia-Shigella*	*S. sonnei/E. fergusonii*	99,78	> 0,05	507	Hg + M
*Escherichia-Shigella*	*S. sonnei/E. fergusonii*	99,78	> 0,05	131	Hg + M
*Neisseria*	*N. cinerea/N. perflava*	99,11	> 0,05	126	Hg
*Escherichia-Shigella*	*S. sonnei/E. fergusonii*	99,78	> 0,05	503	Hg
*Escherichia-Shigella*	*S. sonnei/E. fergusonii*	99,78	> 0,05	109	Hg
*Escherichia-Shigella*	*S. sonnei/E. fergusonii*	99,78	> 0,05	2214	Hg
*Escherichia-Shigella*	*S. sonnei/E. fergusonii*	99,78	> 0,05	154	Hg
*Escherichia-Shigella*	*S. sonnei/E. fergusonii*	99,78	> 0,05	139	Hg
*Delftia*	*D. tsuruhatensis/D. lacustris*	99,78	> 0,05	270	Cd
*Escherichia-Shigella*	*S. sonnei/E. fergusonii*	99,78	> 0,05	1239	Cd
*Escherichia-Shigella*	*S. sonnei/E. fergusonii*	99,78	> 0,05	241	Cd
*Escherichia-Shigella*	*S. sonnei/E. fergusonii*	99,78	> 0,05	107	Cd
*Escherichia-Shigella*	*S. sonnei/E. fergusonii*	99,78	-	-	M
*Escherichia-Shigella*	*S. sonnei/E. fergusonii*	99,78	-	-	M
*Prevotella 7*	*P. melaninogenica*	99,78	-	-	M
*Escherichia-Shigella*	*S. sonnei/E. fergusonii*	99,78	-	-	M
*Escherichia-Shigella*	*S. sonnei/E. fergusonii*	99,78	-	-	M
*Neisseria*	*N. cinerea/N. perflava*	99,11	-	-	M
Uncultured actinobacterium	*V. incomptus*	83,37	-	-	M
*Escherichia-Shigella*	*S. sonnei/E. fergusonii*	99,78	-	-	M

**Fig. 5:**
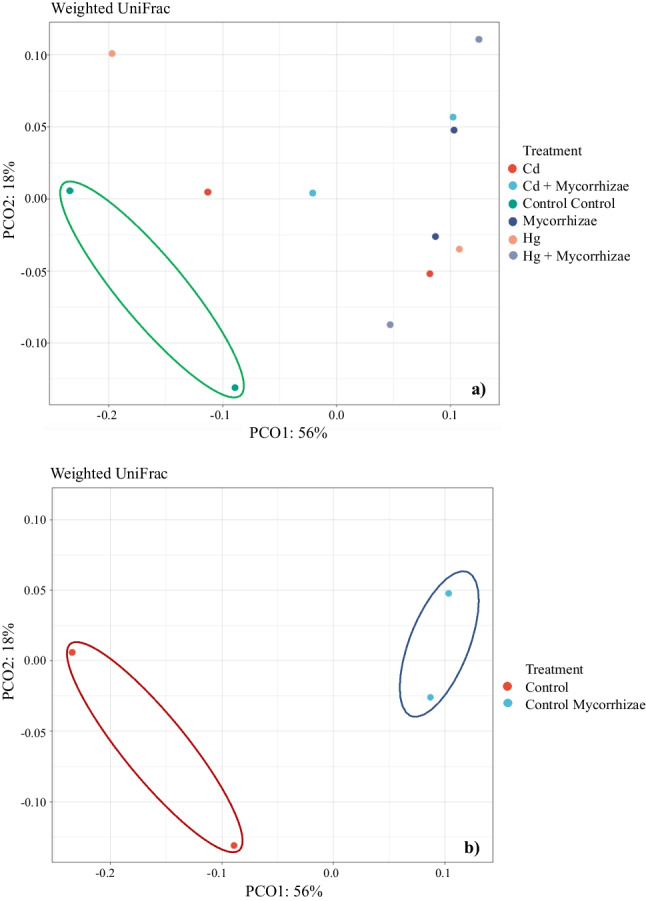
Principal component analysis (PCoA). **a** all treatments and **b** controls

To assess the change in bacterial community structure and composition by beta diversity, a principal component analysis (PCoA) was performed. It has been found that controls without contaminant and mycorrhizae clustered differentially with the rest of the treatments, supporting the idea that microbial diversity is affected by metal contamination despite the bioaccumulative effect of the plant (Fig. 5a).

In the same way, to verify that the addition of mycorrhiza in the soil effectively generates alterations in the microbial community, the analysis was carried out between the control treatment and the control with mycorrhiza, observing considerable changes in beta diversity, confirming the observation in the ASV distribution (Figs. 3 and 5b).

### Relationship between microbiome changes and phytoremediation

The microbial community analyses in the phytoremediation process show that the plant is not carrying out the phytostabilization process alone but is possibly working in consortium with the microbial community present in the soil and that it may be tolerable to metals such as Cd and Hg. A clear example of this is the presence of families such as the *Enterobacteriaceae*, which has been reported by other authors in the biosorption and efflux of metals such as Hg and Cd (Dashti et al. 2019; Hassen et al. 1998; Priyadarshanee et al. 2022). Table 5 shows the remediation mechanisms for metals associated with the microorganisms that have been found in this study and have been reported in the literature. There are some of these microorganisms that have not been reported and were found in almost all the treatments applied, such as *Aguaspirillaceae*, *bacterium ellin*, *Bacteroidetes vadin*, *BIrii41*, *Microscillaceae*, and *Holophaga*, which may possibly be associated with some remediation mechanism. This suggests that they may be involved in the removal process or may only be able to survive in this type of environment. It is evident that except for three families of microorganisms (Table 5), all the other families present a remediation mechanism for the case of Cd, of which the reduction, efflux, secretion, increase the tolerance of the plants can be highlighted, biosorption among others. In the case of Hg, it is evident that only four families have been reported with some mechanism involved in its removal, such as *Streptococcaceae*, *Rhodanobacteraceae*, *Moraxellaceae*, and *Bacillaceae*, with mechanisms such as reduction, efflux, secretion, bioremediation, detoxification, and biosorption (Bae et al. 2002; Baldiris et al. 2018; Chang et al. 2012; Dashti et al. 2019; De et al. 2008; De and Ramaiah 2007; Kinoshita et al. 2013; Pushkar et al. 2021; Seong et al. 2021; Simon et al. 2017; Tuzen et al. 2009; Verma and Kuila 2019).

**Table 5:** Remediation mechanisms for metals associated with microorganisms found in metagenomic analyses

Family name	Metals report	Mechanism involved	Reference
*Aquaspirillaceae*	No reports	-	-
*Bacillaceae*	Cr + 6, Pb, Cd, Hg	Reduction, efflux, secretion	(Baldiris et al. 2018; Banerjee et al. 2019; Dashti et al. 2019; De et al. 2008; De and Ramaiah 2007; Hassen et al. 1998; Priyadarshanee et al. 2022; Pushkar et al. 2021; Shaw and Dussan 2018; Zhu et al. 2019)
*Bacterium Ellin7509*	No reports	-	-
*Bacteroidetes vadinHA17*	No reports	-	-
*BIrii41*	No reports	-	-
*Burkholderiaceae*	Cd, Pb	Increases tolerance in plants (*Brassica campestris* and ryegrass) to pollutants such as Cd; increases Pb accumulation in sorghum	(Ni et al. 2021; Wu et al. 2020, 2019)
*Chitinophagaceae*	Fe, Pb, Cd, Cu	Tolerant	(Giongo et al. 2020; Karray et al. 2020)
*Enterobacteriaceae*	Hg, Cd	Biosorption, efflux	(Dashti et al. 2019; Hassen et al. 1998; Priyadarshanee et al. 2022)
*Gemmatimonadaceae*	Pb, Cd	Stabilization, heavy metals were associated with distinct microbial communities, and these microbes may contribute to the bioremediation of heavy metals	(Chun et al. 2021)
*Holophagaceae*	As, Fe, Sb, Cu, Cd	Might be able to tolerate or metabolize, increases under elevated copper concentrations, tolerant	(Giongo et al. 2020; Sutcliffe et al. 2019; Xu et al. 2020)
*Methylophilaceae*	Cr, Cu, Zn, Cd	High tolerance to metals, microorganism with high potential for soil remediation; presence of this family in areas contaminated by Cd	(Gong et al. 2021; Wang et al. 2016)
*Microscillaceae*	No reports	-	-
*Moraxellaceae*	Cd, Hg	Moraxella sp., a bacterium known to survive in contaminated environments; Cd and Hg bioremediation	(Bae et al. 2002; Verma and Kuila 2019)
*Neisseriaceae*	Pb, Cu, Zn, Cd, Fe	Bioadsorption, tolerant	(Chaturvedi and Archana 2014; Ghimire and McCarthy 2018; Giongo et al. 2020)
*Nitrosomonadaceae*	Cr, Pb, Cd	Metal reduction (Cr), abundant family in environments contaminated by As, Cd, Cr, Ni, Hg	(Caliz et al. 2012; Chen et al. 2018; Chun et al. 2021; Drewniak et al. 2016)
*Pirellulaceae*	Cd	Reduction	(Dai et al. 2020)
*Prevotellaceae*	Cd	Cd resistance	(Ramírez-Acosta et al., 2021)
*Rhodanobacteraceae*	Hg	Bacteria with mechanisms for the detoxification of Hg, grow in environments with Hg. Hg resistance genes	(Seong et al. 2021; Simon et al. 2017)
*Rhodothermaceae*	Co, Ni, As	Metabolism of metals	(Cerqueda-García et al. 2020; Gu et al. 2017)
*Sphingobacteriaceae*	Cr + 6, Pb, Cd	Biosorption, reduction	(Chun et al. 2021; Prabhakaran et al. 2019; Pushkar et al. 2021)
*Streptococcaceae*	Cd, Cr, As, Hg, CH_3_Hg	Biosorption	(Chang et al. 2012; Kinoshita et al. 2013; Tuzen et al. 2009)
Uncultured actinobacterium	Heavy metals in general	Bioadsorption, plant growth, helps plants withstand higher metal stress; Pb, Zn, Cr, Cd, Cu, As, and Ni	(Bankar and Nagaraja 2018; El Baz et al. 2015)
Uncultured *Holophaga* sp.	No reports	-	-
*Xanthomonadaceae*	Cr + 6, Cu, Fe, Pb	Reduction, tolerant	(Baldiris et al. 2018; Giongo et al. 2020; Pushkar et al. 2021)

The results demonstrate that the root microbiome, both without and with mycorrhiza, plays an important role in promoting plant growth to improve yield and may also regulate soil fertility, as other authors have argued (Sharaff et al. 2020; Yadav et al. 2020). It is important then to understand the microbiome of *L. perenne* in the remediation processes of soils contaminated with Cd and Hg, to increase removal efficiencies. Therefore, the application of mycorrhizae considerably improves the removal efficiency of these metals and redistributes them in the different parts of the plot, increasing the percentages of soil removal. These microbiomes demonstrated that they have the capacity to promote plant growth and rise Cd and Hg removal directly or indirectly through the release of hormones or the release of organic or enzymatic nutrients and the supply of nutrients (Kumar et al. 2019; Yadav et al. 2021).

## Conclusions

The results show that adding mycorrhizae to the phytoremediation process with *L. perenne* considerably favors the absorption and distribution of both metals in the structural parts of the plant (shoot and root). However, it has been observed that mycorrhizae stimulate a better microbiome-plant interaction, improving the concentration of metal removed in the soil and increasing the diversity of families of microorganisms in the soil.

It has been shown that up to 75% of the families of microorganisms detected have been reported in different studies involving some mechanism of contaminant removal. Examples of this are stabilization and biosorption, which help increase metal tolerance in plants and in many cases increase metal accumulation. In this study, it was found that the addition of mycorrhizae favors this increase in the accumulation of both metals. Some other microorganisms have not been reported to have a mechanism involving metal removal processes, but in this study, they have been identified, as is the case of *Aquaspirillaceae*, *Bacterium Ellin7509*, *Bacteroidetes vadinHA17*, *BIrii41*, *Microscillaceae*, and *Holophaga* sp. that it is recommended for further studies to carry out tests of mechanisms involved in the removal of metals, especially Hg and Cd.

The results have shown that the root microbiome, both mycorrhizal and mycorrhizal, plays an important role in plant growth, improving yield and regulating soil fertility.

## Data Availability

The bacterial 16S rRNA gene amplicon sequencing data obtained in this study have been deposited under NCBI BioProject ID accession number PRJNA837400.
